# Comparison of factors that affect the quality of life of Korean land police officers and maritime police officers

**DOI:** 10.3389/fpsyt.2025.1517012

**Published:** 2025-04-24

**Authors:** Seung-Woo Han, Hyun-Seok Yoon

**Affiliations:** ^1^ Department of Nursing, Kwangju Women’s University, Gwangju, Republic of Korea; ^2^ Division of Police Administration, Kwangju Women’s University, Gwangju, Republic of Korea

**Keywords:** quality of life, leisure activities, job satisfaction, self-esteem, police

## Abstract

**Background:**

The quality of life (QoL) of land police officers (LPOs) and maritime police officers (MPOs), involves not only their work life but also their personal life, including their interactions with family and friends.

**Methods:**

This study was conducted with LPOs and MPOs based in Region J of South Korea. Data were collected from September 1 to September 30, 2024. Instruments used included measures of job satisfaction, leisure satisfaction, self-esteem, and overall quality of life. Data were analyzed using stepwise multiple regression.

**Results:**

For LPOs, the factors that significantly affect the QoL were subjective health status (‘moderate’, β = -0.213, *p* = .001; ‘poor’, β = -0.130, *p* = .021), job satisfaction (β = 0.414, *p* = .001) and leisure satisfaction (β = 0.393, *p* = .001). These variables explained 64.2% of the variance in QoL. For MPOs, the factors that significantly affect the QoL were low economic level (β = -0.440, *p* = .007), job satisfaction (β = 0.269, *p* = .001), leisure satisfaction (β = 0.488, *p* = .001), and self-esteem (β = 0.164, *p* = .006). These variables explained 53.2% of the variance in QoL.

**Conclusion:**

The findings of this study indicate a clear need to implement counseling interventions to improve the quality of life of LPOs and MPOs. Additionally, to improve their overall quality of life, programs to enhance physical and mental health must be developed.

## 1 Introduction

In recent years, the crime rate has increased sharply, and the crimes have become increasingly vicious and more directly threatening to the lives and property of the public. In addition to their fundamental duties, the roles of land police officers (LPOs) are increasingly complicated by new threats from a growing number of accidents and disasters (1). While performing their duties, LPOs are exposed to significant physical and mental risks that can precipitate conditions such as post-traumatic stress disorder, depression, and anxiety, which can challenge their mental health (2–4).

Marine police officers (MPOs) encounter unique challenges in their line of duty. They manage high-risk operations like interdicting illegal fishing vessels, responding to oil spills, and assisting distressed ships (5). The officers also face high noise levels, extreme temperature fluctuations, and frequent shift changes, which can significantly contribute to fatigue and stress (6).

In contemporary society, demands of citizens for quality of life (QoL) and safety are increasingly emphasized, so the importance of the role of LPOs is increasing. Furthermore, the standard of happiness at the individual level has shifted from materialism to intrinsic forms of satisfaction such as QoL and well-being (6). As the emphasis on individual happiness and QoL grows, the public’s demand for safety and security is also expected to increase continually. Exploring the QoL of LPOs is crucial, because it not only contributes to their well-being but also to the overall safety of the nation and the satisfaction of its citizens.

QoL represents an individual’s perceived level of satisfaction and happiness with their current life circumstances, and is closely associated with psychological well-being. Therefore, to improve QoL, efforts must extend across multiple criteria (7). This study explores the complex factors that influence the QoL of LPOs, by applying the bottom-up spillover theory, which is a specific approach within the wider spillover theory of happiness (8).

As mentioned above, since the work environments and roles of LPOs and MPOs are different, it will be difficult to consistently understand the quality of life of LPOs and MPOs. Of course, the difference between the two groups may be large, small, or non-existent, but verifying the difference could be useful data for welfare policies for the two occupations, effective personnel allocation, and police organization reorganization. In addition, police officers’ job satisfaction and quality of life can affect not only their work performance but also the quality of police services provided to the public. Police officers are under high stress due to the nature of their work, so there is a need to explore strategies to improve psychological well-being through leisure satisfaction and self-esteem.

In general discussions about QoL, research often contrasts bottom-up spillover theory with top-down spillover theory. Bottom-up spillover theory highlights the role of individuals’ experiences with external environments in shaping their QoL, whereas top-down spillover theory emphasizes individuals’ intrinsic dispositions, and implies that internal characteristics primarily drive perceptions of QoL (9).

According to bottom-up spillover theory, the QoL is determined by the aggregation of positive and negative experiences and their utility in everyday life. This approach posits that satisfaction or positive experiences in daily activities contribute to increased utility across major life domains (10). For instance, among LPOs, experiences that increase job satisfaction or leisure satisfaction in their daily lives to good levels can contribute to improvement in overall QoL. This possibility exemplifies a practical application of bottom-up spillover theory; i.e., that improvements in specific areas of daily life can lead to general enhancements in QoL (9). Therefore, this study selected job satisfaction and leisure satisfaction from bottom-up spillover theory, and self-esteem, an intrinsic disposition, from the top-down spillover theory as the key variables for research.

Prior studies have predominantly focused on individuals who work in emergency settings, and have specifically addressed PTSD comparisons between POs and firefighters (11), as well as between POs and emergency medical personnel (12). However, comparative studies between LPOs and MPOs have been notably absent. This research undertakes a direct comparison between land and maritime police to identify organizational challenges and to contribute to improving both the work environment and the QoL of these officers.

In particular, unlike other Western countries, Korea is adjacent to China, Japan, and North Korea by sea on three sides, so in addition to the maritime police’s own duties (rescue, territorial sea security, etc.), its main duties are maritime enforcement, such as maritime military response and prevention of illegal activities at sea (13). In a previous study (14), Korean maritime police officers were found to be higher than land police officers in both stress and depression. Along with land police officers, maritime police officers should not let down their guard against high incidence of accidents and sudden events. Therefore, considering Korea’s geopolitical characteristics, this study compares the factors influencing the quality of life of land and marine police officers and seeks ways to improve their quality of life.

Building on the foundations of both the bottom-up and top-down spillover theories, this study aims to identify factors that influence QoL in these two distinct groups of law-enforcement personnel. The goal is to exploit these insights to develop programs to improve the QoL of POs, and thereby contribute to systemic improvements that support their physical and mental health. In addition, the results of this study will serve as important basic data for comparing the quality of life of land and coast police officers in maritime countries adjacent to the continents of Europe, Africa, Europe and Asia, similar to Korea.

### 1.1 Purpose of the study

The primary aim of this study was to identify factors that influence the QoL among Korean land LPOs and MPOs. Specifically, the study seeks to:

- Assess levels of job satisfaction, leisure satisfaction, self-esteem, and overall QoL among the participants.
- Identify variations in job satisfaction, leisure satisfaction, self-esteem, and QoL based on the general characteristics of the participants.
- Explore the correlations among these variables.
- Identify factors that significantly affect the participants’ QoL.

## 2 Materials and methods

### 2.1 Research design

This investigation was a descriptive survey study aimed at assessing the factors that affect the QoL of Korean LPOs and MPOs.

### 2.2 Study subjects

The participants in this study were full-time POs employed at police stations, district offices, and patrol stations located in Gwangju, Jeonnam, Jeonbuk, and the West Regional Headquarters Korea Coast Guard of South Korea.

The sampling area for this study is area J, adjacent to the west coast of Korea. This area is adjacent to China, and the intensity of the Coast Guard’s work is considerably greater than in other areas due to the crackdown on illegal fishing. However, because the Coast Guard located on the east coast mainly focuses on maritime security and the intensity of work may be lower than that of police in other regions and on land, this study selected police stations located in region J. Officers who had served for less than one year or those who declined to participate in the study were excluded from the sample. The sample size was determined using the G*Power 3.1.9.4 program, calculating for a significance level of 0.05, a power of 0.95, an effect size of 0.10, and twelve predictor variables. This calculation resulted in a total of 270 participants, and accounting for an anticipated dropout rate of 20%, questionnaires were distributed to 324 officers. Out of the 324 questionnaires returned, 14 were discarded due to insincere responses. The final sample for analysis consisted of 310 participants.

### 2.3 Research tools

#### 2.3.1 Job satisfaction

To assess job satisfaction, we used the Korea Minnesota Satisfaction Questionnaire (K-MSQ), which was developed by Dawis and Lofquist (15) at the Minnesota Institute of Industrial Relations and translated by Park (16) to confirm its validity and reliability. The K-MSQ is divided into three subscales; i.e., extrinsic satisfaction, intrinsic satisfaction, and overall satisfaction, and consists of a total of 20 items. A representative survey question measures the level of satisfaction with “being able to work using my abilities.” Each item is scored on a 5-point Likert scale from 1 ‘very dissatisfied’ to 5 ‘very satisfied’, so scores increase as job satisfaction increases. In this study, K-MSQ had Cronbach’s *α* = .901.

#### 2.3.2 Leisure satisfaction

To assess leisure satisfaction, we used the Leisure Satisfaction Scale (LSS) (17), which has good reliability and validity (18). The LSS is organized into six subscales: psychological, educational, social, relaxation, physical, and aesthetic, and consists of a total of 24 items. A representative survey question is “My leisure activities help me learn about other people.” Each item on the scale is rated using a 5-point Likert scale that ranges from 1 ‘not at all’ to 5 ‘very much so’, so scores increase as satisfaction from leisure activities increases. In this study, LSS had Cronbach’s *α* = .852.

#### 2.3.3 Self-esteem

To assess self-esteem, we used the self-esteem scale developed by Rosenberg (19) and translated by Jeon (20). The scale is composed of 10 items, evenly split between five positively-worded and five negatively-worded statements. A representative survey item is “I think I am a valuable person like other people.” It uses a 5-point Likert scale, ranging from 1 ‘not at all’ to 5 ‘very much so’. For analytical purposes, responses to negative items are reverse-scored, to ensure consistent scoring across all items. The total score increases as self-esteem increases. In this study, this scale had Cronbach’s *α* = .825.

#### 2.3.4 Quality of life

To asses QoL, Min et al. (22) developed the first WHOQOL-BREF based on the existing WHOQOL-BREF (21) developed by WHO. This study used the developed tool, which was finally modified by Kang (23) to fit the Korean style. The QoL scale consists of 24 items divided into four domains: physical health with eight items, psychological health with four items, social relationships with six items, and environmental factors with six items. A representative survey question is “To what extent do I feel that my life is meaningful?” Each item is assessed using a 5-point Likert scale, ranging from 1 ‘not at all’ to 5 ‘very much so’. The score increases as perceived QoL increases. In this study, this instrument had Cronbach’s α = .802.

### 2.4 Data collection

This study received approval from the head of the police department following a review of the consent forms and questionnaires. The data collection period was set from September 1 to September 30, 2024.

The process involved contacting police departments, police stations, and district offices located in Gwangju, Jeonnam and Jeonbuk, and the West Regional Headquarters Korea Coast Guard. Following approval from department heads, study descriptions, consent forms, and questionnaires were mailed out. The information sheet and consent form provided details on the study’s purpose and procedures, assured anonymity and confidentiality, and highlighted the participants’ right to withdraw at any time. Each police station head informed their officers about the study. The whole process typically took 15 min. The completed questionnaires were sealed and collected by the researcher either in person or by mail. All participants received a small token(chocolate bars and simple refreshments) of appreciation for their involvement.

### 2.5 Data analysis

Data collected from the study were analyzed using SPSS/WIN 23.0 software.

Means and standard deviations were calculated for job satisfaction, leisure satisfaction, self-esteem, and QoL both among LPOs and among MPOs. To analyze how general characteristics affected differences in QoL, t-tests, and one-way ANOVA were used. Correlations among study variables were determined using Pearson’s correlation coefficient *r*. Stepwise multiple regression analysis was utilized to identify factors that significantly affect the QoL of the participants.

### 2.6 Ethical considerations

In order to protect the life and human rights of the research subjects, approval was obtained from the K University Institutional Review Board(IRB No. 202412-HR-001-32). Before the survey, written consent for participation in the study was obtained from the research participants, and then data were collected through anonymous self-administration through a structured questionnaire. The researcher explained to the subjects that they can stop completing the questionnaire at any time if discomfort or harm is discovered through the study, that the subjects’ information obtained through this study will be kept strictly confidential and will be used only for research purposes, and that the subject’s personal information will not be revealed for any reason or form. The time required to fill out the survey was 15 to 20 minutes, and those who responded to the survey were given a small gift.

## 3 Research

### 3.1 Differences in QoL by general characteristics

Age: Among the LPOs, the largest age group was ‘40s’ (n=58, 36.3%), whereas for MPOs, the largest age group was ‘30s’ (n=67, 44.7%). Marital Status: Most respondents were ‘married’ (n=115 LPOs, 71.9%; n=83 MPOs, 55.3%). Education: Most respondents were ‘college graduates’ (n=90 LPOs, 56.3%; n=79 MPOs, 52.7%). Religion: The most common response regarding religion was ‘none’ (n=103 LPOs, 64.4%; n=111 MPOs, 74.0%). Years of Service: Among the LPOs, the most common tenure was ‘21 years or more’ (n=50, 31.3%); for MPOs, it was ‘5 years or less’ (n=60, 40.0%). Rank: The most common rank among LPOs was ‘lieutenant’ (n=58, 36.3%), whereas for MPOs, it was ‘captain’ (n=37, 24.7%).

Subjective Health Status: ‘Medium’ was the most-reported health status (n=114 LPOs, 71.3%; n=87 MPOs, 58.0%). Economic Level: ‘Medium’ was also the most common economic level (n=128 LPOs, 80.0%; n=111 MPOs, 74.0%). Salary Satisfaction: ‘Medium’ was also the most common assessment of satisfaction (n=113 LPOs, 70.6%; n=85 MPOs, 56.7%).

Factors that significantly affected QoL differed between the two groups. For LPOs, the significant factors were subjective health status (F = 12.179, *p* <.001), economic level (F = 3.451, *p* = .034), and salary satisfaction (F = 6.296, *p* = .002). For MPOs they were marital status (F = 3.397, *p* = .036), economic level (F = 6.041, *p* = .003), and salary satisfaction (F = 3.773, *p* = .025) (**Table 1**).

**Table 1:** Quality of life by general characteristics.

Variable	Category	n(%)		LPOs (n=160)			n(%)		MPOs (n=150)		
				M(SD), t or F (*p*)					M(SD), t or F (*p*)		
Age	20s	22	(13.8)	3.36	(.50)	.424 (.736)	24	(16.0)	3.40	(.45)	.179 (.910)
	30s	32	(20.0)	3.29	(.34)		67	(44.7)	3.39	(.52)	
	40s	58	(36.3)	3.33	(.43)		42	(28.0)	3.34	(.47)	
	Over 50s	48	(30.0)	3.41	(.58)		17	(11.3)	3.31	(.56)	
Marital status	Unmarried^a^	42	(26.3)	3.34	(.42)	1.348 (.263)	65	(43.3)	3.32	(.48)	3.397 (.036) c<ab
	Married^b^	115	(71.9)	3.36	(.49)		83	(55.3)	3.42	(.50)	
	Other(bereavement, divorce, separation)^c^	3	(1.9)	2.92	(.26)		2	(1.3)	2.58	(.59)	
Educational background	High school	30	(18.8)	3.30	(.43)	.717 (.543)	36	(24.0)	3.34	(.63)	.551 (.648)
	College	29	(18.1)	3.27	(.50)		31	(20.7)	3.29	(.43)	
	University	90	(56.3)	3.37	(.41)		79	(52.7)	3.42	(.45)	
	Graduate or higher	11	(6.9)	3.48	(.85)		4	(2.7)	3.28	(.42)	
Religion	Have	57	(35.6)	3.32	(.44)	-.472 (.638)	39	(26.0)	3.42	(.41)	.861 (.392)
	None	103	(64.4)	3.36	(.49)		111	(74.0)	3.35	(.52)	
Working Period	Less than 5 years	28	(17.5)	3.39	(.49)	1.042 (.387)	60	(40.0)	3.37	(.52)	.094 (.984)
	6-10 years	27	(16.9)	3.29	(.33)		34	(22.7)	3.34	(.50)	
	11-15 years	28	(17.5)	3.23	(.40)		21	(14.0)	3.34	(.45)	
	16-20 years	27	(16.9)	3.32	(.42)		21	(14.0)	3.39	(.50)	
	More than 21 years	50	(31.3)	3.44	(.57)		14	(9.3)	3.42	(.51)	
Rank	Constable	17	(10.6)	3.42	(.55)	1.985 (.099)	35	(23.3)	3.26	(.52)	1.526 (.198)
	Senior Patrol Officer	17	(10.6)	3.33	(.38)		37	(24.7)	3.33	(.57)	
	Assistant Inspector	31	(19.4)	3.22	(.37)		30	(20.0)	3.55	(.42)	
	Inspector	58	(36.3)	3.30	(.44)		31	(20.7)	3.35	(.43)	
	Senior Inspector or higher	37	(23.1)	3.51	(.56)		17	(11.3)	3.38	(.48)	
Subjective health status	Upper^a^	34	(21.3)	3.67	(.54)	12.179 (*p*<.001) cb<a	51	(34.0)	3.42	(.44)	2.557 (.081)
	Medium^b^	114	(71.3)	3.27	(.39)		87	(58.0)	3.38	(.52)	
	Low^c^	12	(7.5)	3.14	(.57)		12	(8.0)	3.07	(.51)	
Economic level	Upper^a^	7	(4.4)	3.57	(.54)	3.451 (.034) c<a	6	(4.0)	3.78	(.42)	6.041 (.003) cb<a
	Medium^b^	128	(80.0)	3.38	(.48)		111	(74.0)	3.41	(.44)	
	Low^c^	25	(15.6)	3.15	(.31)		33	(22.0)	3.15	(.61)	
Salary satisfaction	Upper^a^	5	(3.1)	3.67	(.62)	6.296 (.002) c<a	8	(5.3)	3.59	(.40)	3.773 (.025) c<a
	Medium^b^	113	(70.6)	3.41	(.48)		85	(56.7)	3.44	(.44)	
	Low^c^	42	(26.3)	3.15	(.35)		57	(38.0)	3.23	(.56)	

a,b,c = Scheffe test.

### 3.2 Job satisfaction, leisure satisfaction, self-esteem, and QoL

For LPOs, average job satisfaction was 3.30 ± 0.53 out of a possible 5, whereas for MPOs it was 3.32 ± 0.59; the difference is not significant. Leisure satisfaction was equal for both groups, averaging 3.62 ± 0.64 for LPOs and 3.62 ± 0.61 for MPOs. Mean self-esteem scores were 3.57 ± 0.49 for LPOs, and 3.06 ± 0.32 for MPOs. Mean QoL ratings were 3.35 ± 0.47 for LPOs and 3.37 ± 0.50 for MPOs. Only self-esteem differed significantly between the groups (**Table 2**).

**Table 2:** Differences in job satisfaction, leisure satisfaction, self-esteem, and quality of life.

	LPOs (n=160)		MPOs (n=150)		*t*	*p*
	M(SD)		M(SD)			
Job Satisfaction	3.30	(.53)	3.32	(.59)	-.352	.725
Leisure Satisfaction	3.62	(.64)	3.62	(.61)	-.117	.907
Self-Esteem	3.57	(.49)	3.06	(.32)	11.108	.001
QoL	3.35	(.47)	3.37	(.50)	-.352	.725

### 3.3 Correlations among variables

LPOs demonstrated significant positive correlations between QoL and job satisfaction (r = 0.709, p < 0.001), leisure satisfaction (r = 0.691, *p* <.001), and self-esteem (r = 0.362, *p* <.001). MPOs also demonstrated positive correlations between QoL and job satisfaction (r = 0.529, *p* <.001), leisure satisfaction (r = 0.613, *p* <.001), and self-esteem (r = 0.277, *p* = .001) (**Table 3**).

**Table 3:** Correlation among QoL, Job Satisfaction, Leisure Satisfaction, Self-Esteem.

	QoL	
	LPOs (n=160)	MPOs (n=150)
	r (*p*)	r (*p*)
Job Satisfaction	.709 (*p*<.001)	.529 (*p*<.001)
Leisure Satisfaction	.691 (*p*<.001)	.613 (*p*<.001)
Self-Esteem	.362 (*p*<.001)	.277 (.001)
QoL	1	1

### 3.4 Factors that influence QoL

To examine the factors that affect the QoL of participants, statistically significant variables from demographic characteristics were converted to dummy variables. For LPOs, these factors were subjective health status, economic level, and salary satisfaction; for MPOs, they were marital status, economic level, and salary satisfaction. Subsequently, a multiple regression analysis was performed, incorporating job satisfaction, leisure satisfaction, and self-esteem. Durbin-Watson test (24) residuals gave *d* = 1.974 for LPOs and 1.912 for MPOs; both are close to 2, which indicates lack of first-order autocorrelation.

Analysis did not detect multicollinearity among the effects of the variables. Calculated tolerance limits ranged from 0.103 to 0.767 for LPOs and from 0.123 to 0.911 for MPOs; all exceed the threshold of 0.1, and therefore indicate lack of multicollinearity. The Variance Inflation Factor (VIF) values ranged from 1.304 to 9.114 for LPOs and from 1.097 to 8.113 for MPOs; all are well below the critical value of 10, and therefore confirm the lack of multicollinearity among the variables.

The multiple regression models were significant for both LPOs (F = 32.662, *p* <.001) and MPOs (F = 19.661, *p* <.001). For LPOs, QoL was significantly affected by subjective health status ‘medium’ (β = -0.213, *p* = .001) and ‘low’ (β = -0.130, *p* = .021), job satisfaction (β = 0.414, *p* = .001), and leisure satisfaction (β = 0.393, *p* = .001); the explanatory power of these variables was 64.2%. For MPOs, QoL was significantly affected by economic level ‘low’ (β = -0.440, *p* = .007), job satisfaction (β = 0.269, *p* = .001), leisure satisfaction (β = 0.488, *p* = .001), and self-esteem (β = 0.164, *p* = .006); the explanatory power of these variables was 53.2% (**Table 4**).

**Table 4:** Factors affecting quality of life.

Variable	B	SE	β	t	*p*
LPOs (n=160)					
(Constant)	1.081	.279		3.880	.001
Subjective health status (ref: Upper)					
Medium	-.221	.061	-.213	-3.596	.001
Low	-.231	.099	-.130	-2.329	.021
Economic level (ref: Upper)					
Medium	-.066	.125	-.056	-.524	.601
Low	-.128	.143	-.099	-.894	.373
Salary satisfaction (ref: Upper)					
Medium	.207	.152	.201	1.360	.176
Low	.138	.161	.130	.860	.391
Job Satisfaction	.366	.054	.414	6.707	.001
Leisure Satisfaction	.289	.045	.393	6.487	.001
Self-Esteem	.023	.052	.024	.436	.663
MPOs (n=150)					
(Constant)	.650	.368		1.768	.079
Marriage (ref: Unmarried)					
Married	.081	.059	.081	1.368	.174
Etc	-.146	.254	-.034	-.574	.567
Economic level (ref: Upper)					
Medium	-.349	.177	-.310	-1.967	.051
Low	-.525	.190	-.440	-2.759	.007
Salary satisfaction (ref: Upper)					
Medium	.041	.155	.041	.266	.790
Low	.088	.163	.086	.540	.590
Job Satisfaction	.229	.058	.269	3.955	.001
Leisure Satisfaction	.401	.053	.488	7.614	.001
Self-Esteem	.253	.091	.164	2.769	.006

## 4 Discussion

This study was conducted to compare and analyze job satisfaction, leisure satisfaction, self-esteem, and QoL among Korean LPOs and MPOs, and to identify the factors that influence their QoL.

The findings revealed that job satisfaction significantly influences the QoL for both groups of officers. These results align with prior research (1) that has shown the influence of job satisfaction on the QoL among shift-working POs. Job satisfaction has been found to significantly affect QoL across various professions, especially those that require specialized knowledge and a commitment to human life and safety, such as occupational therapists (25) and staff in psychiatric or hospital intensive care units (26). Similarly, another previous study found that when police officers had high coworker support at work, their PTSD symptoms were significantly reduced and it was beneficial to their mental health (27). Therefore, in order to improve job satisfaction, support systems such as coworker support must be implemented first, and ways to utilize social support within the workplace must be continuously sought. LPOs and MPOs, due to their significant authority and responsibility for public safety and life, are profoundly affected if job satisfaction is not achieved. The bottom-up spillover theory states that life satisfaction is affected by various life domain satisfaction measures and sub-factors (28). In general, this is consistent with research results showing that workers’ life satisfaction is influenced by work (work relationships, working hours). This is consistent with the research results (29) that workers’ life satisfaction is generally affected by work (satisfaction with work relationships, satisfaction with working hours). We posit that this lack of satisfaction could significantly threaten not only their QoL but also their physical and mental health. Future research should explore various aspects of the work environment that could affect QoL, including not only job satisfaction but also satisfaction with the job environment.

This study found that leisure satisfaction significantly influences the QoL of LPOs and MPOs. Previous research (30) have emphasized physical and mental well-being as the most crucial elements of QoL, and have noted that leisure satisfaction enhances psychological, social, and environmental interests, and thereby offers benefits, such as improved stress management, that can benefit mental health (31). As mentioned earlier, the bottom-up spillover theory states that life satisfaction is influenced by measuring satisfaction in various areas of life, one of which is leisure, which is consistent with this study (29). Particularly for LPOs and MPOs, who are more susceptible to burnout compared to other professions, the continuous nature of burnout in a demanding, unpredictable 24-hour work environment, in which where opportunities for recovery are scarce, has been shown to further degrade QoL (32, 33). In Korea, the 40-hour workweek system was implemented nationwide in July 2007 with the enforcement of regulations on working hours for full-time police officers (34). As leisure time expanded and perceptions of leisure were changing positively due to the overall reduction in working hours, expectations for the use of leisure were rising. Future research should investigate various environmental factors that can increase leisure satisfaction among LPOs and MPOs, and thereby promote their physical and mental well-being, which are the most crucial elements of QoL. Programs that are developed must be compatible with practical application and use in their actual work environments.

This study identified self-esteem as a significant factor influencing QoL, with notable effects specifically among MPOs. MPOs, distinct from their land counterparts, face unique geographical and environmental challenges and dangers associated with coastal areas. They report experiencing psychosocial stress as well as problems related to separation from families, and to loneliness aboard ships (35). Top-down theories are the strategies that people generally use to optimize their subjective well-being. It was said that overall self-esteem affects domain-specific self-esteem (top-down) (36). A recent study (37) revealed that Korean MPOs have higher rates of depression compared to LPOs and firefighters. This frustration of fundamental psychological needs not only results in elevated stress but also diminishes self-esteem following the recognition of job-related problems (38, 39). Another study (40) highlighted self-esteem as a protective factor that can mitigate negative emotional states such as depression and burnout among POs. The results (41) of a study on the self-esteem of police officers in Korea showed that police officers are an occupational group with higher self-esteem than other occupational groups due to the nature of their jobs, and that the higher their self-esteem, the more they tend to accept incidents positively and actively try to solve problems. Such high self-esteem is believed to have had a positive impact on the quality of life in this study due to its inherent psychological characteristics of leading daily life in a more positive and active direction. Future research should focus on self-esteem. It should also explore other variables like resilience and self-efficacy, which are crucial for promoting mental well-being, which is a significant component of QoL. Additionally, the reasons that they differently affect the two groups should be identified.

The current study identified that subjective health status was a significant determinant of QoL only among LPOs. The findings demonstrate that a ‘moderate’ or ‘lower’ subjective health status significantly reduces QoL. Prior research (42) has confirmed subjective health status as a significant determinant of QoL, and that poor subjective health contributes to decreased QoL (43). The 24-hour on-call work patterns of land-based POs pose multiple threats to their physical and mental health. LPOs frequently encounter physical threats in the field, whereas MPOs typically serve in static, long-duration postings at sea. This difference may contribute to the more substantial impact on subjective health status of LPOs compared to MPOs. Future research should delve into the subjectively perceived health status of both LPOs and MPOs, examining it from both physical and mental perspectives to better understand its effects on their QoL.

Lastly, economic level was identified as a factor that influences QoL, but was statistically significant only among MPOs. This study found that a ‘lower’ economic level decreases QoL. This finding aligns with previous research (44), which indicated that low household income reduces QoL among Koreans. This is consistent with the results (45) of a study targeting African Americans in the United States, which showed that low income is associated with low quality of life. We speculate that this relationship may affect QoL of MPOs more than of LPOs because Korean Coast Guard MPOs officers are restricted to government-stipulated salaries, and being at sea, do not have possibility of additional income. This income dissatisfaction may combine with challenging work environment factors such as geographic isolation and family separation to reduce QoL. Future research should examine the effects of specific aspects of the work environment, such as working conditions and satisfaction with compensation, to assess their contributions to QoL.

This study of Korean LPOs and MPOs has identified that job satisfaction and leisure satisfaction significantly increased the QoL of both groups, but that the ‘medium’ and ‘low’ subjective health status decreased the QoL of LPOs, whereas self-esteem increased the QoL of MPOs and low economic status reduced it. By identifying the distinctions between these effects on the QoL of the two groups, the results may suggest ways to increase their QoL and, by extension, their physical and mental health, and thereby ultimately contribute to national safety and security.

### 4.1 Limitations of the study and implications for research and practice

Firstly, this study considered LPOs and MPOs from only Area J, so the generalizability of the findings to the QoL of the entire police force may be limited. Future research should broaden the scope to include additional comprehensive demographic characteristics, such as variations in regional populations and geographical areas. Second, as a result of this study, in the Working Period part, ‘LP0’ had 50 people over 21 years, and ‘MPO’ had 14 people, showing that the number of samples between the two groups was not properly adjusted. Since these differences not only affect the actual research results but can also lead to statistical bias, we will make sure to clarify statistical control through appropriate sample distribution in future studies. Additionally, the quantitative nature of this research may not fully capture the nuanced experiences of the QoLs of LPOs and MPOs. Future studies should employ qualitative methods to phenomenologically investigate the lived experiences and the inherent meanings of QoL among LPOs and MPOs. Lastly, this research was confined to variables such as job satisfaction, leisure satisfaction, and self-esteem. Future studies should incorporate a broader array of variables that could affect QoL, considering various factors that LPOs and MPOs encounter in their professional duties and daily lives, to more effectively explore the actual determinants of QoL.

## 5 Conclusion

This study was designed to identify factors that affect the QoL of LPOs and MPOs, and to compare the differences. The goal was to provide foundational data for the development of programs that can increase the physical and mental health, and overall QoL of LPOs and MPOs.

The findings indicate that for LPOs, job satisfaction, and leisure satisfaction increased their QoL, whereas ‘medium’ and ‘low’ subjective health status decreased it. For job satisfaction, leisure satisfaction, and self-esteem increased QoL, whereas for MPOs, ‘low’ economic level decreased it. Building on these results, future research should investigate additional factors that may influence the physical and mental well-being of LPOs and MPOs, such as happiness and well-being, which are beyond the traditional measures of QoL. Additionally, efforts to improve the QoL for both LPOs and MPOs should be supported by a conducive work environment and institutional backing that allows them to effectively safeguard public life and safety.

## Data Availability

The original contributions presented in the study are included in the article/supplementary material. Further inquiries can be directed to the corresponding author.
